# Dissecting the Mechanisms of Left Ventricular Diastolic Dysfunction and Inflammation in Peritoneal Dialysis Patients

**DOI:** 10.1371/journal.pone.0062722

**Published:** 2013-05-13

**Authors:** Cho-Kai Wu, Yin-Tsen Huang, Heng-Hsu Lin, Chung-Yi Yang, Yu-Chung Lien, Jen-Kuang Lee, Jenq-Wen Huang, Kuan-Yu Hung

**Affiliations:** 1 Division of Cardiology, Department of Internal Medicine, National Taiwan University College of Medicine and Hospital, Taipei, Taiwan; 2 Graduate Institute of Clinical Medicine, College of Medicine, National Taiwan University, Taipei, Taiwan; 3 Department of Family Medicine and Health Management Center, Far Eastern Memorial Hospital, New Taipei City, Taiwan; 4 Division of Cardiology, Department of Internal Medicine, Far Eastern Memorial Hospital, New Taipei City, Taiwan; 5 Department of Medical Imaging, National Taiwan University College of Medicine and Hospital, Taipei, Taiwan; 6 Division of Nephrology, Department of Internal Medicine, National Taiwan University College of Medicine and Hospital, Taipei, Taiwan; 7 Department of Laboratory Medicine, National Taiwan University Hospital, Taipei, Taiwan; 8 Department of Clinical Pathology, Far Eastern Memorial Hospital, New Taipei City, Taiwan; 9 Graduate Institute of Biomedical Electronics and Bioinformatics, National Taiwan University, Taipei, Taiwan; National Taiwan University Hospital, Taiwan

## Abstract

**Objective:**

Patients with symptoms of heart failure and preserved left ventricular (LV) systolic function are commonly encountered in clinical practice especially in peritoneal dialysis (PD) patients. We hypothesized that adiposity might influence LV diastolic function through systemic inflammation in this specific group.

**Methods:**

We designed a cross-sectional study in 173 prevalent PD patients. LV diastolic dysfunction was diagnosed by echocardiography. PD patient without LV diastolic dysfunction served as the control group. Serum inflammatory biomarkers were examined including tissue necrosis factor-alpha (TNF-α) and interleukin-6 (IL-6). The location and amount of adipose tissue were assessed by computerized tomography (CT) at the level of the fourth lumbar vertebra.

**Results:**

Subjects with LV diastolic dysfunction had higher levels of the pro-inflammation cytokines and more visceral and peritoneal fat (all P<0.001) than control subjects. A significant correlation was found between visceral adipose tissue and pro-inflammatory cytokines (r = 0.70; P<0.001). Multivariable regression analysis found that the relationship between visceral adipose tissue and LV diastolic dysfunction became insignificant when either TNF-α or IL-6 were introduced into the model, although TNF-α and IL-6 were both significantly associated with LV diastolic dysfunction even after adjusting for visceral fat (OR = 1.51; 95% CI = 1.09–2.02; P = 0.033 and OR = 1.62; 95% CI = 1.09–1.82; P = 0.031, respectively).

**Conclusions:**

Larger amounts of adipose tissue were associated with higher serum pro–inflammatory levels in PD patients, which might be related to the development of LV diastolic dysfunction. Modulating inflammatory reactions in PD patients can be a useful therapeutic approach for managing LV diastolic dysfunction.

## Introduction

Left ventricular (LV) diastolic dysfunction has become an increasing concern in recent years. Studies suggest that at least one–third of patients with congestive heart failure have diastolic heart failure (DHF) despite having normal or near normal LV ejection fractions [1]. In patients with complex comorbidities, LV diastolic dysfunction could be an independent prognostic marker for patients with preserved LV contractility [2]. Patients with chronic kidney disease (CKD) stage 5 suffer from fluid overload and have a high prevalence of hypertension and LV hypertrophy (which is a physiological response to pressure and volume overload). Together, these factors contribute to the high prevalence of LV diastolic dysfunction in patients with CKD stage 5 [3], [4]. Currently, there is scant information describing the mechanisms of LV diastolic dysfunction in end-stage renal disease (ESRD) patients.

Inflammation and oxidative stress are reportedly associated with the high rate of cardiovascular events in hemodialysis and continuous ambulatory peritoneal-dialysis (PD) patients alike [5]–[8]. Our recent studies found that the correlation between inflammatory cytokines and LV diastolic dysfunction in PD patients was significantly stronger than in subjects with normal creatinine levels [9]. It is possible that systemic inflammation could impact LV diastolic dysfunction via a number of different mechanisms. For example, inflammatory cytokines may cause cardiac diastolic dysfunction by decreasing diastolic calcium re-uptake by downregulating sarcoplasmic reticulum Ca^2+^-ATPase gene expression [10]. Alternatively, high levels of inflammatory cytokines may even influence LV diastolic dysfunction directly by altering calcium handling protein concentration and function.

The distribution of body fat, which is modified in PD patients play a pivotal role in the development and progression of both diastolic and systolic heart failure [11]. A recent study suggested that weight reduction using formula diet might improve renal function in obese patients [12]. Taking the above points into accounts, PD patients have both higher inflammation and abnormal fat distribution which are both important factors for the development of LV diastolic dysfunction. For searching possible resolve for the clinical pivotal issue, LV diastolic dysfunction, it's crucial to clarify the relationship between inflammation, central adiposity and LV diastolic function in the distinct population. Consequently, the purpose of this study was to investigate the association between the above factors in PD patients using current golden standard for central obesity and LV diastolic function. According to our previous work [9], [10], serum tissue necrosis factor-alpha (TNF-α) and Interleukin-6 (IL-6) had a synergistic effect with PD to induce LV diastolic dysfunction. The level of the above two pro-inflammatory cytokines were much higher than the controls comparing with other inflammatory cytokines [9] and in addition, the effects of the cytokines over the expression of sarcoplasmic reticulum Ca^2+^-ATPase protein, which is a major determinants for LV diastolic dysfunction are more apparently than the conventional inflammatory cytokines, such as C-reactive protein. Therefore, we chose TNF-α and IL-6 as our targeting inflammatory markers in current study.

## Materials and Methods

### Ethics statement

We certify that all the applicable institutional and governmental regulations concerning the ethical use of human volunteers were followed during this research. Written informed consent was obtained from every participating subject, and the study was approved by the institutional review board of the National Taiwan University Hospital.

### Study participants and study design

**Figure 1:**
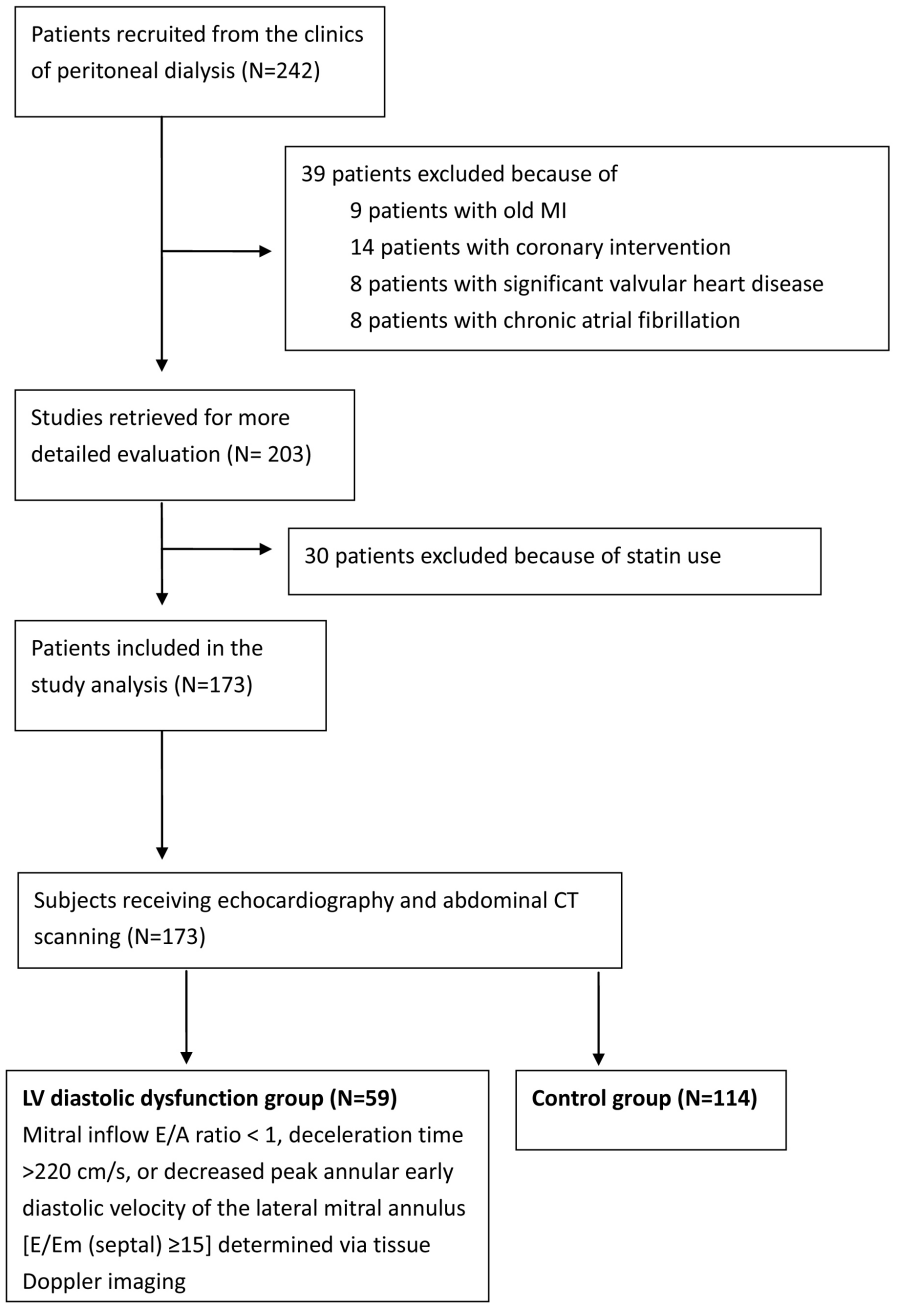
The designed patient flow diagram.

Between July 2007 and March 2009, 242 homogenous Taiwanese patients aged over 20 who had received PD catheter surgery and PD with a conventional glucose-based lactate-buffered PD solutions (UltraBag; Baxter Healthcare SA, Singapore) for >6 months at the special clinics of peritoneal dialysis of the National Taiwan University Hospital were consecutively enrolled. Patients with hypertension, diabetic and chronic glomerular nephritis would also be included (Table 1). Patients with hepatic disease, a history of myocardial infarction, coronary intervention, cardiac myopathy, pericardial disease, or significant valvular heart disease (≥moderate), chronic obstructive pulmonary disease, chronic atrial fibrillation, clinical signs of acute infection, or other chronic inflammatory conditions were excluded. Patients who received statins, lipid lowering agents and/or other medication that could potentially influence plasma inflammatory cytokine levels were also excluded. The designed patient flow diagram was depicted in Figure 1. Finally 173 PD patients were included in the final analysis. All subjects underwent echocardiography and abdominal computerized tomography (CT) scanning. In total, 59 participants were classified as having LV diastolic dysfunction as defined by a mitral inflow early rapid filling wave (E) divided by peak velocity of the late filling wave due to atrial contraction (A) ratio < 1, a deceleration time (DT) >220 cm/s, or decreased peak annular early diastolic velocity of the lateral mitral annulus (Em)[E/Em (septal) ≥15] determined via tissue Doppler imaging [13]–[15]. The stage of diastolic dysfunction would be clarified according to the recommendations of European Association for Echocardiography and the American Society of Echocardiography [16]. Briefly, grade I was defined as mild diastolic dysfunction with the mitral E/A ratio is < 0.8 and DT is >220 cm/s; grade II was defined as pseudonormal pattern with the mitral E/A ratio is 0.8 to 1.5 and E/Em ratio is 9 to 12; grade III was defined as severe diastolic dysfunction with an average E/Em ratio is >12. The remaining 114 subjects without LV diastolic dysfunction served as the control group.

**Table 1:** Baseline patient demographics, echocardiographic characteristics, and adipose tissue measurements of the 173 subjects included in the study.

	Control Subjects	Case Subjects	
Baseline characteristics	(n = 114)	(n = 59)	P
Age (years)	54.3±8.6	57.5±7.2	0.015
Women (n)	42	25	0.51
Smokers (n)	34	15	0.59
HTN (n)	68	45	0.04
DM (n)	42	28	0.19
Fasting plasma glucose (mg/dL)	96.2±19.5	97.3±11.8	0.69
Total cholesterol (mg/dL)	187.3±41.7	194.4±33.9	0.26
HDL (mg/dL)	46.8±12.3	49.6±9.8	0.14
LDL (mg/dL)	122.9±23.5	129.3±32.3	0.13
TG (mg/dL)	125.3±85.3	142.4±96.1	0.23
Height (cm)	163±5.9	162±6.5	0.30
Body weight (kg)	64±6.3	69±7.2	0.0002
BMI (kg/m^2^)	24±3.6	26±3.1	0.0004
TNF-α (pg/nL)	8.8±3.9	15.6±5.3	<0.0001
IL-6 (pg/nL)	21.6±4.2	36.2±3.8	<0.0001
HbA1c (%)	5.73±0.72	5.91±0.81	0.13
HOMA	2.31±1.24	2.44±1.31	0.52
Primary renal diagnosis			
DM	39	23	0.61
CGN	34	15	0.59
Others	41	21	0.87
Time on PD (hr)	18.9±1.6	19.1±1.3	0.46
Kt/V	1.72±0.34	1.91±0.25	0.57
Residual renal function (ml/min)	1.94±0.31	2.31±0.22	0.25
**Echocardiographic characteristics**			
Interventricular septum thickness	9.1±1.1	9.6±1.9	0.029
LV posterior wall thickness	9.6±1.2	10.2±1.6	0.006
LV end diastolic diameter	47.2±5.6	49.8±6.3	0.009
LV end systolic diameter	29.2±4.6	31.2±3.7	0.004
LA diameter	32.6±6.5	37.3±5.8	<0.0001
LV ejection fraction	64.4±7.9	66.2±9.1	0.17
E	79.3±12.3	75.6±15.6	0.08
A	58.6±15.8	85.5±14.1	<0.0001
E/A	1.43±0.49	0.91±0.33	<0.0001
DT	198.1±42.6	231.4±49.8	<0.0001
Em	11.6±3.2	7.3±2.9	<0.0001
E/Em	7.2±3.5	12.1±3.8	<0.0001
LV mass index	48.1±26.7	42.2±27.2	0.18
Grade I diastolic dysfunction	0	27	–
Grade II diastolic dysfunction	0	23	–
Grade III diastolic dysfunction	0	9	–
**Adipose tissue distribution**			
Total fat (cm^2^)	242.7±87.2	318.6±95.1	<0.0001
Subcutaneous fat (cm^2^)	172.6±64.1	179.2±67.4	0.52
Visceral fat (cm^2^)	70.1±32.9	139.4±47.3	<0.0001
Peritoneal fat (cm^2^)	41.9±28.3	74.6±32.8	<0.0001
Retroperitoneal fat (cm^2^)	29.20±17.77	64.8±28.6	<0.0001
Visceral/subcutaneous fat ratio	0.41±0.31	0.77±0.43	<0.0001

Data are presented as mean ± standard deviation unless otherwise indicated.

Abbreviations: BMI, body mass index; WC, waist circumflex; DM, diabetes; HTN, hypertension; HOMA, homeostasis model of insulin resistance; TNF-α, tissue necrosis factor-alpha; IL-6, interleukin-6; LDL, low density lipoprotein; HDL, high density lipoprotein; TG, triglyceride; PD, peritoneal dialysis; kt/v, k- dialyzer clearance of urea, t-dialysis time, v-volume of distribution of urea, approximately equal to patient's total body water; LA, left atrial diameter; LV, left ventricular; E, mitral inflow E wave; A, mitral inflow A wave; DT, mitral inflow deceleration time; Em, Peak annular early diastolic velocity of the lateral mitral annulus in tissue Doppler imaging; IVRT, interventricular relaxation time.

### Biochemical data

Blood samples were collected from the antecubital vein between 8:00 AM and 10:00 AM from all patients in a supine or sitting position following 12 hr of fasting. Plasma glucose and serum total cholesterol (TC), high-density lipoprotein cholesterol (HDL), low-density lipoprotein cholesterol (LDL), and triglycerides (TG) were determined using an automatic analyzer (Toshiba TBA 120FR, Toshiba Medical Systems Co., Ltd., Tokyo, Japan). Plasma insulin levels were measured using an AxSYMautoanalyzer (Abbott Laboratories, Abbott Park, IL, USA), and insulin resistance was assessed by calculating the homeostasis model of insulin resistance (HOMA-IR) using the following formula: [fasting glucose (mmol/L) ×

fasting insulin (μ U/mL)]/22.5 [17]. Serum IL-6 was measured using a high-sensitivity enzyme-linked immunoassay (ELISA, R&D Systems, Minneapolis, MN, USA), which had a minimal detectable range of 0.016 to 0.110 pg/mL. Serum tissue TNF-α was assayed in duplicate using a Quantikine HS/human TNF-α immunoassay (R&D Systems, Minneapolis, MN, USA), which had a minimal detectable dose range of 0.038 to 0.191 pg/mL. The calculated overall inter- and intra-assay coefficients of variation were: 6.5–9.6% and 6.9–7.8% for IL-6 and 7.4–10.6% and 3.1–8.5% for TNF-α, respectively. All the sample was processed in a blind fashion with examiners who were not aware the clinical characteristics of the patients.

### Echocardiography

Left atrial (LA) diameter, LV end diastolic diameter, systolic diameter, interventricular septum thickness, LV posterior wall thickness, E/A ratio and DT were measured according to the American Society of Echocardiography guidelines using a Vivid 7 Dimension echocardiography (GE Health care, Chalfont St. Giles, UK). LV ejection fraction was calculated as previously described [18]. The LV mass index was calculated from the LV end diastolic diameter and systolic diameter, interventricular septum thickness, and LV posterior wall thickness according to the method of Devereux et al. [19]. Peak annular early and late diastolic velocity of the lateral mitral annulus in tissue Doppler imaging (Em and Am) were also recorded. Doppler and color Doppler studies were performed to detect valvular heart disease. Significant valvular heart disease was defined as at least moderate aortic or mitral stenosis/regurgitation.

### Determination of adipose tissue distribution by CT

Imaging of each subject was performed using a 16-MDCT scanner (LightSpeed 16, GE Healthcare, Milwaukee, WI, USA). Image analysis software (ImageJ, version 1.43q; National Institutes of Health, Bethesda, MD, USA) was employed with an attenuation range of −50 to −250 Hounsfield units to quantify subcutaneous, visceral, and total abdominal adipose tissue areas on a single cross-sectional image obtained at the level of the umbilicus with the summation of adipose tissue in 5mm thickness [20]. Results were expressed in cm^2^. The visceral/subcutaneous adipose tissue area ratio (V/S ratio) was subsequently calculated. The intra- and inter-reader reproducibility for current study is 91.2 to 97.4% and 89.7 to 95.5% respectively for measurement of visceral fat, subcutaneous fat, peritoneal fat and retroperitoneal fat measurements after repeat measurements for 50 patients.

### Statistical Analysis

Analyses were performed using SPSS 16.0 for Windows XP (SPSS Inc. Chicago, IL, USA). To test whether the data were normally distributed, the Kolmogorov-Smirnov test was applied. All the continuous variables were normally distributed. Therefore, between group baseline characteristics and echocardiographic findings were compared using the Student's unpaired t test for continuous data and a chi-square test for categorical data. Comparisons of mean values across groups and correlations between continuous variables were assessed via linear regression. Odds ratios (ORs) and 95% confidence intervals (CIs) were estimated by logistic regression. Associations between cytokines and Doppler parameters were calculated with Pearson's correlation coefficient. To assess the association between adipose tissue, serum biomarkers, and LV diastolic dysfunction, multivariable models were applied. Covariates that were associated with LV diastolic dysfunction such as age, gender, hypertension, smoking, and body mass index (BMI) were incorporated. All p values were two-sided and p values <0.05 were considered statistically significant. We used two sample average method of the DSS research power calculator to estimate the required sample size (http://www.dssresearch.com/KnowledgeCenter/toolkitcalculators/samplesizecalculators.aspx). Based on our previous study, if PD patients with LV diastolic function had an TNF-α level of 15.7±4.8 and patients otherwise had an TNF-α level of 10.4±4.2, [9] we estimated that 10 patients in either group would be needed to achieve 80% statistical power (given α = 0.05).

## Results

### Demographics and echocardiographic data in PD patients

Of the 173 PD subjects consecutively enrolled in this study, 59 were allocated to the LV diastolic dysfunction group, leaving 114 subjects to serve as the control group. Baseline characteristics of the study participants included in the two groups have been summarized in Table 1. Subjects with LV diastolic dysfunction were predominantly older, female, and suffered more frequently from hypertension and hyperlipidemia. PD patients with LV diastolic dysfunction also had greater body masses, larger waist circumferences, and significantly higher TNF-α and IL-6 levels.

Compared to the control group, subjects with LV diastolic dysfunction had a larger end-diastolic LV volume and end-LV systolic volume (p = 0.009 and p = 0.004), greater thickness of both end-diastolic septum and posterior wall (both p< 0.05), and a larger left atrial diameter at end-systole (p<0.0001). Patients with LV diastolic dysfunction also had a prolonged DT (p<0.0001), a decreased mitral inflow E/A ratio (p<0.0001), and a decreased Em (p<0.0001) compared to the PD patients. In addition, subjects allocated to the LV diastolic dysfunction group had significantly higher total amount of fat (measured in cm^2^), including visceral fat, peritoneal fat, and retroperitoneal fat (all p<0.0001) than subjects included in the control group.

### Correlation of adipose tissue distribution and clinical parameters in PD patients

Pearson bivariate correlation tests between total fat, visceral fat, V/S ratio, and biochemical and echocardiographic parameters were performed. Age, body mass index (BMI), HOMA, and TG all had significant low to moderate correlations with visceral fat (r = 0.52, p<0.0001; r = 0.65, p<0.01; r = 0.42, p = 0.001; and r = 0.37, p = 0.006, respectively, as described in Table 2). Markers of inflammation, TNF-alpha and IL6, were both strongly correlated with the amount of visceral fat (r = 0.73, p<0.0001 and r = 0.75, p<0.0001, respectively). HDL was negatively correlated with visceral fat (r = −0.33, p<0.0001). Among the echocardiographic characteristics, LA diameter had a moderate association with visceral fat (r = 0.56, p<0.0001) and the various diastolic function parameters, including DT, E/A and E/Em, were all significantly associated with visceral fat (r = 0.28, p = 0.023; r = −0.37, p = 0.002; and r = 0.36, p = 0.004, respectively).

**Table 2:** Pearson bivariate correlations between adipose tissue and various clinical characteristics.

	Total Fat		Visceral Fat		V/S fat ratio	
	Coefficient	P	Coefficient	P	Coefficient	P
Age	0.33	0.23	0.52	<0.0001	0.48	<0.0001
BMI	0.73	<0.0001	0.65	<0.0001	0.24	0.008
HOMA	0.48	<0.0001	0.42	0.001	0.15	0.23
TC	0.07	0.40	0.05	0.51	0.11	0.89
HDL	−0.32	<0.0001	−0.33	<0.0001	−0.122	0.004
LDL	0.11	0.24	0.21	0.33	0.18	0.41
TG	0.21	0.03	0.37	0.006	0.27	0.009
TNF-α	0.59	<0.0001	0.73	<0.0001	0.63	<0.0001
IL-6	0.66	<0.0001	0.75	<0.0001	0.68	<0.0001
LA diameter	0.39	0.001	0.56	0.0007	0.33	0.004
LV ejection fraction	0.18	0.15	0.25	0.09	0.22	0.18
DT	0.25	0.039	0.28	0.023	0.21	0.029
E/A	−0.36	0.002	−0.37	0.002	−0.31	0.007
E/Em	0.21	0.09	0.36	0.004	0.31	0.008

Abbreviations: BMI, body mass index; HOMA, homeostasis model of insulin resistance; TC, total cholesterol; LDL, low-density lipoprotein; HDL, high-density lipoprotein; LA, left atrial/atrium; LV, left ventricular; E, mitral inflow E wave; A, mitral inflow A wave; Em, Peak annular early diastolic velocity of the lateral mitral annulus in tissue Doppler imaging.

### Correlation of plasma levels of TNF-α, IL-6, and LV diastolic dysfunction parameters in PD patients

**Figure 2:**
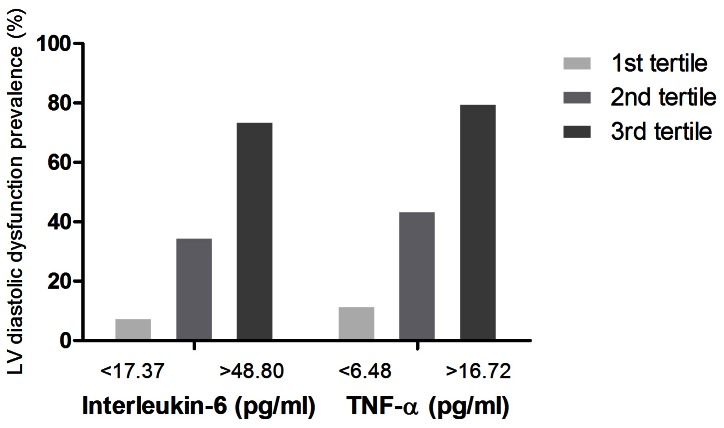
The prevalence of left ventricular (LV) diastolic dysfunction in PD patients divided into tertiles based on interleukin-6 (IL-6) and tumor necrosis factor-α (TNF-α) levels.

**Figure 3:**
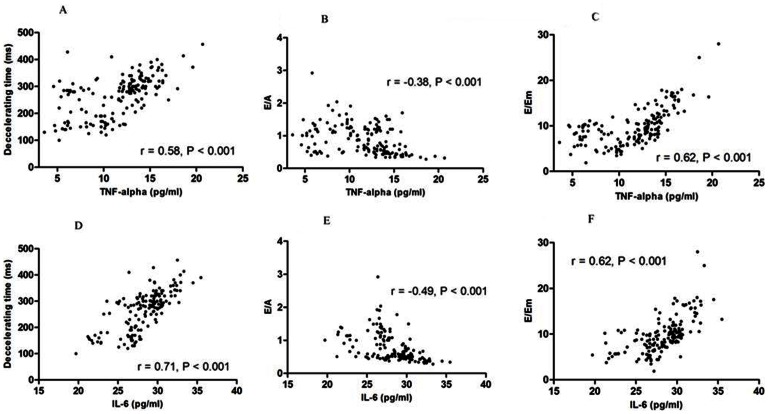
Correlation between interleukin-6 (IL-6), tissue necrosis factor-alpha (TNF-α), and echocardiographic diastolic function parameters in PD patients. (A) TNF-α and mitral valve ejection flow deceleration time (DT, r = 0.58, P<0.001); (B) TNF-α and the ratio of mitral valve ejection flow (E) divided by mitral valve atrium flow (A, r = −0.38, P<0.001); (C) TNF-α and the ratio of E divided by early diastolic lengthening velocities in tissue Doppler imaging (Em, r = 0.62, P<0.001); (D) IL-6 and DT (r = 0.71, P<0.001); (E) IL-6 and E/A (r = −0.49, P<0.001); and (F) IL-6 and E/Em (r = 0.62, P<0.001).

PD patients were divided into tertiles based on TNF-α and IL-6 levels [TNF-α (pg/ml): group 1, <6.48; group 2, 6.48∼16.72; group 3, >16.72 and IL-6 (pg.ml): group 1, <17.37; group 2, 17.37∼48.80; group 3, >48.80]. A significant difference in the distribution of patients with LV diastolic dysfunction was noted for both serum pro-inflammatory cytokine levels (Figure 2). Bivariate correlations of LV diastolic function parameters and plasma levels of the pro-inflammatory cytokines have been illustrated in Figures 3A–F. Both pro-inflammatory cytokines correlated with all the LV diastolic dysfunction parameters significantly in PD patients. We also calculated the association correlation between TNF-α and IL-6. The correlation between TNF-alpha and IL-6 was significant(r = 0.72, p = 0.002). However, the inflammation biomarkers, IL-6 and TNF-alpha, didn't correlate with systolic dysfunction. The correlation between TNF-alpha, IL-6 and LV ejection fraction were r = 0.26, p = 0.45 and r = 0.37, p = 0.32 respectively. Besides, the residual renal function had no significant correlation with diastolic function parameter for E/A, DT and E/Em (r = 0.23, p = 0.55; r = 0.36, p = 0.49; r = 0.29, p = 0.61 respectively).

### Association between LV diastolic dysfunction, inflammation and adipose tissue in PD patients

Pro-inflammation cytokine levels of TNF-α and IL-6 were higher in subjects with LV diastolic dysfunction. To adjust for the confounding effect of other clinical factors, multivariable analysis was performed. TNF-α and IL-6 were still associated with LV diastolic dysfunction after adjusting for age, gender, BMI, diabetes mellitus, hypertension, HOMA, TNF-α or IL6, LDL, and LV mass index (OR = 1.78; 95% CI = 1.15–2.59; p = 0.003 for TNF-α and OR = 1.93; 95% CI = 1.18–2.73, p = 0.002 for IL-6) as described in Table 3 and Table 4 (model 1).

**Table 3:** Multivariable logistic regression models for parameters associated with left ventricular diastolic dysfunction.

	Odds ratio	95% confidence interval	P
**Model 1**			
Age	1.18	1.02–1.32	0.045
Gender	0.7	0.22–1.18	0.19
BMI	1.12	0.53–1.83	0.54
DM	2.1	0.81–3.32	0.42
HTN	2.9	0.77–9.62	0.11
HOMA	0.78	0.32–1.37	0.19
TNF-α	1.78	1.15–2.59	0.003
LDL	1.05	0.81–1.34	0.67
LV mass index	1.21	0.79–1.63	0.74
**Model 2**			
Visceral fat	1.41	1.16–1.77	0.023
**Model 3**			
Peritoneal fat	1.58	1.2–1.97	0.017
**Model 4**			
Retroperitoneal fat	1.37	0.92–1.88	0.08
**Model 5**			
Visceral fat	1.27	0.69–1.92	0.27
TNF-α	1.51	1.09–2.02	0.033
**Model 6**			
Peritoneal fat	1.26	0.75–1.89	0.37
TNF-α	1.39	1.05–1.78	0.041

Models 2–4 adjust for age, gender, BMI, DM, HTN, HOMA, and LDL. Models 5 and 6 additionally adjust for tissue necrosis factor-alpha.

Abbreviations: BMI, body mass index; DM, diabetes mellitus; HTN, hypertension; HOMA, homeostasis model of insulin resistance; TNF-α, tissue necrosis factor-alpha; LDL, low-density lipoprotein; LV, left ventricular.

**Table 4:** Multivariable logistic regression models for parameters associated with left ventricular diastolic dysfunction.

	Odds ratio	95% confidenceinterval	P
**Model 1**			
Age	1.24	1.09–1.45	0.041
Gender	0.7	0.27–1.24	0.18
BMI	1.1	0.51–1.92	0.59
DM	2.3	0.79–3.49	0.48
HTN	2.8	0.72–8.18	0.24
HOMA	0.75	0.37–1.42	0.28
IL-6	1.93	1.18–2.73	0.002
LDL	1.06	0.79–1.38	0.62
LV mass index	1.28	0.74–1.83	0.68
**Model 2**			
Visceral fat	1.41	1.16–1.77	0.023
**Model 3**			
Peritoneal fat	1.58	1.2–1.97	0.017
**Model 4**			
Retroperitoneal fat	1.37	0.92–1.88	0.08
**Model 5**			
Visceral fat	1.21	0.72–1.83	0.39
IL-6	1.62	1.09–1.82	0.031
**Model 6**			
Peritoneal fat	1.18	0.75–1.62	0.32
IL-6	1.47	1.11–1.92	0.037

Models 2–4 adjust for age, gender, BMI, DM, HTN, HOMA, and LDL. Models 5 and 6 additionally adjust for interleukin-6.

Abbreviations: BMI, body mass index; HOMA, homeostasis model of insulin resistance; DM, diabetes mellitus; HTN, hypertension; LV, left ventricular; IL-6, interleukin-6; LDL, low-density lipoprotein.

Multivariable regression analysis was subsequently performed for fat distribution and its association with LV diastolic dysfunction in PD patients. Only visceral fat (adjusted OR = 1.41; 95% CI = 1.16–1.77; p = 0.023) and peritoneal fat (adjusted OR = 1.58; 95% CI = 1.2–1.97; p = 0.017) were associated with the development of LV diastolic dysfunction as described in Table 3 and 4, models 2–4. Multivariable analysis was repeated and the impact of fat was abolished after either of the pro-inflammatory cytokines was included in the analysis. The effect of TNF-α remained significant (OR = 1.51; 95% CI = 1.09–2.02; p = 0.033 and OR = 1.39; 95% CI = 1.05–1.78; p = 0.041, respectively after controlling for visceral fat or peritoneal fat) as described in Table 3, models 5 and 6. Similar results were also observed for IL6 (OR = 1.62; 95% CI = 1.09–1.82; p = 0.031 and OR = 1.47; 95% CI = 1.11–1.92; p = 0.037, respectively after controlling for peritoneal fat or visceral fat).

## Discussion

In this study, we found that PD patients with LV diastolic dysfunction had significantly higher levels of pro-inflammatory cytokines. In addition, a significant association between LV diastolic dysfunction and inflammatory cytokine levels was noted in this specific group of patients. In PD patients with higher levels of pro-inflammatory cytokines, the relationship between fat distribution, inflammation, and LV diastolic dysfunction was examined. We also adjusted factors associated with LV diastolic dysfunction using several logistic regression models. These analyses showed that certain fat deposits might act partly through inflammation to affect LV diastolic dysfunction in PD patients.

Immuno-inflammatory activation plays a pivotal role in the development and in the progression of heart failure by some studies [11]. Inflammatory cytokines reportedly contribute to LV remodeling and to the progression of heart failure by influencing cardiac contractility, inducing hypertrophy, and promoting myocyte apoptosis and fibrosis [21], [22]. Although there do not appear to be any published studies that report a precise prevalence rate of diastolic heart failure in PD patients, a higher prevalence of LV diastolic heart failure in ESRD patients than in the general population is expected in light of the occurrence of inflammation, fluid overload, hypertension, renin-angiotensin-aldosterone system activation, and LV hypertrophy [4], [22]. Our previous work also revealed that systemic inflammation was associated with the development of LV diastolic dysfunction in PD patients [9].

To the best of our knowledge, this is the first study to address the relationship between adipose tissue contents and diastolic dysfunction in PD patients. Elevated or excessive amounts of adipose tissue are regarded as risk factors for a number of diseases such as insulin resistance, type 2 diabetes mellitus, and atherosclerosis, which can lead to major cardiovascular sequelae. Further, adipose tissue is associated with changes in both inflammatory cells and biochemical markers of inflammation. For example, the levels of circulating fibrinogen and other acute phase reactants are elevated in obese patients [23], [24]. In the current study, we demonstrated that inflammation plays a pivotal role in the development of LV diastolic dysfunction rather than adipose tissue itself in PD patients. In this study, both intra-abdominal and subcutaneous adipose tissue was measured in PD patients by CT, which is regarded as a sensitive and accurate method [25]. Therefore, the independent effect of obesity for LV diastolic dysfunction may be less in PD patients than the general population.

In our recent study examining inflammation and LV diastolic dysfunction in PD patients [9], the role of pro-inflammatory cytokines in a group of PD patients and controls was assessed. That study identified a significant correlation between LV diastolic dysfunction and serum TNF-α, and IL-6 levels in PD patients and an interaction between PD and inflammation. TNF-α was also shown to aggravate LV diastolic dysfunction. Together with the current study findings, even the adipose tissue loads in PD patients might increase a patient's susceptibility to LV diastolic dysfunction at least partly through inflammation. Therefore, anti-inflammatory therapy might be an effective treatment for PD patients with evidence of systemic inflammation and LV diastolic dysfunction. Statins (3-hydroxy-3-methylglutaryl coenzyme A reductase inhibitors) are a family of lipid-lowering drugs known to exert several effects beyond the lipid-lowering effects. In addition to being an effective lipid-lowering agent for PD and hemodialysis patients with dyslipidemia, several studies have also documented that statins could decrease serum cytokine levels in CKD patients [26], [27] In a previous study regarding statin for CKD patients, there was no significant reduction in cardiovascular and all-cause mortality [28]. It is important to recognize that the subjects in the previous study were not limited to those with a higher inflammatory status. On the other hand, previous studies for statin over CKD patients never measured the changes of LV diastolic function as a primary outcome. Future studies could also focus on the improvement of LV diastolic function after controlling serum inflammatory cytokines levels, especially in PD patients.

The present study showed that adipose tissue may be associated with LV diastolic dysfunction partly through the effect of inflammation in PD patients. There exerts some explanations. First, a recent report demonstrated that inflammation can reduce myocardial function by inhibiting myocyte contractility and by remodeling the extracellular matrix [29]. Second, transgenic mice with overexpressing cardiac TNF-α would first develop myocardial hypertrophy with preserved systolic function [30]. Additionally, a number of clinical studies have found that cytokine levels in cardiac tissue were higher in subjects with preserved systolic function but with a diseased myocardium resulting from hypertrophic cardiomyopathy, aortic stenosis, or transplant myocardium [31], [32]. It is well-known that a higher amount of visceral adipose tissue may produce higher levels of inflammatory cytokines and may in turn, lead to the development of LV diastolic dysfunction.

In our current studies, we showed that both total fat and visceral fat had a strong correlation with inflammation which is consisted with previous study [22]. However, the inflammation markers did not have a significant correlation for subcutaneous adipose tissue and retroperitoneal fat volumes measurements and had a moderate significant correlation with peritoneal fat volume (r = 0.56, p<0.001)(data not shown). According to a recent study in PD patients, associations between fat accumulations may be a potent exacerbating factor of inflammation in this population, especially visceral fat [33]. Therefore, although PD might lead to abnormal fat distribution, visceral fat could still present for the severity of inflammation to some extent.

Our study had limitations; first, we only use cross-sectional data to infer longitudinal relationship. Although a model is identifiable, more other associations and possible pathways may be demonstrated and a precise causal relationship should be relied on further prospective studies. Second, the patients included in our current study are all Taiwanese. The generalizability to other population should be proved by further study. Third, besides TNF-α and IL-6 we did not measure additional pro-inflammatory cytokines that were not easily discerned in usual clinical practice. In addition, since the actual duration of ESRD or PD was missed in some patients, we could not correlate the relationship between the duration of PD with circulating biomarkers or diastolic dysfunction.

## Conclusion

In conclusion, for the first time, we delineated the complex relationship between adipose tissue accumulation, inflammation, and LV diastolic dysfunction in PD patients. The strength of this study is in its precise definitions. We measured central obesity by CT and defined LV diastolic dysfunction by a combination of variable echocardiographic parameters. This study demonstrated that a greater amount of visceral adipose tissue was associated with inflammation, which was related to subclinical LV diastolic dysfunction in PD subjects.
